# Phytochemical compositions of extract from peel of hawthorn fruit, and its antioxidant capacity, cell growth inhibition, and acetylcholinesterase inhibitory activity

**DOI:** 10.1186/s12906-017-1662-y

**Published:** 2017-03-11

**Authors:** Panpan Wu, Fajie Li, Jianyong Zhang, Bin Yang, Zhaojie Ji, Weidong Chen

**Affiliations:** 1 Anhui University of Chinese Medicine, Hefei, 230012 China; 2 Institute of Chinese Materia Medica, China Academy of Chinese Medical Sciences, No. 16 Nanxiaojie, Dongzhimennei Ave., Dongcheng District, Beijing, 100700 China; 3 State Key Laboratory of Dao-di Herbs, National Resource Center for Chinese Materia Medica, China Academy of Chinese Medical Sciences, Beijing, 100700 China; 4 Flow Station of Post-Doctoral Scientific Research, China Academy of Chinese Medical Sciences, Beijing, 100700 China; 5 Pharmacy School, Zunyi Medical University, Guizhou, 563000 China

**Keywords:** Hawthorn peel, Macroporous resin extract, Phytochemical composition, Antioxidant capacity, Cell growth inhibition, AChE inhibitory activity

## Abstract

**Background:**

Hawthorn fruit (HF) is a well-known traditional medicine in China with the effects of improving digestion and regulating qi-flowing for removing blood stasis. Modern pharmacological experiments showed that HF extract has various pharmaceutical properties and flavonoids are considered as the main bioactive compounds. In this paper, Diaion HP-20 adsorption chromatography was used to enrich flavonoids in PHF, and the phytochemical composition of EPHF was analyzed by high performance liquid chromatography (HPLC) and liquid chromatography tandem mass spectrometry (LC-MS). In addition, EPHF’s antioxidant capacity, acetylcholinesterase (AChE) inhibitory activity and cytotoxic activity were evaluated.

**Methods:**

EPHF was obtained by Diaion HP-20 adsorption chromatography. Phytochemical composition of EPHF was analyzed qualitatively and quantitatively using HPLC and LC-MS. Radical scavenging capacity of EPHF was estimated using 2,2-diphenyl-1-picryhydrazyl (DPPH) assay and oxygen radical absorbance capacity (ORAC) assay. The AChE inhibitory activity of EPHF was evaluated by Ellman method. Cytotoxic activity of EPHF was assessed by means of MTT assay.

**Results:**

Eight kinds of components were identified, in which ideain with the value of 179.4 mg/g was identified to be present in the highest level in EPHF, followed by (−)-epicatechin, chlorogenic acid, cyanidin 3-arabinoside, hyperoside and isoquercitrin at the concentrations of 40.9, 10.0, 1.4, 0.4 and 0.2 mg/g, respectively. The contents of these compounds in EPHF were much higher than those in PHF and HF. In addition, EPHF exhibited strong antioxidant and AChE inhibitory activity (ORAC value: 11.65 ± 2.37 μM Trolox equivalents (TE)/mg, DPPH IC_50_ value: 6.72 μg/mL, anti-AChE activity IC_50_ value: 11.72 μg/mL) compared with PHF and HF. Moreover, EPHF exhibited high levels of cytotoxicity on MCF-7 and SKOV-3 human tumour cell lines in a dose-dependent manner with the IC_50_ of 2.76 and 80.11 μg/mL, respectively.

**Conclusions:**

Macroporous resin is useful for the extraction and separation of the total flavonoids from PHF. The contents of flavonoids especially anthocyanin in EPHF were increased significantly compared with the PHF, and EPHF exhibited strong antioxidant, AChE inhibitory activity and cytotoxicity on human tumour cells.

## Background

Medicinal plants are one of the most important sources of medicinal agents and their beneficial healing effects have been well demonstrated in ancient human civilizations worldwide. Hawthorn is distributed in the Northern Hemisphere mostly in China, Europe and North America [1] and has a long history of usage as a traditional medicine. In Europe and America, the extract of hawthorn leaves and flowers (eg. WS 1442, LI 132) are used for the treatment of hypertension and heart disease [2–4]. In China, hawthorn fruit (HF) has the effects of improving digestion and regulating qi-flowing for removing blood stasis. HF not only commonly be used as a traditional digestant, but also be combined with other Chinese medicines to treat cardiovascular diseases, for example, HF and Danshen (the root of *Salvia miltiorrhiza* Bge.) are used together to treat coronary disease and hyperlipemia. Modern pharmacological experiments showed that HF extract has anti-tumour, antioxidant [5], anti-aging and prevention of Alzheimer’s effects [6–8]. HF extract also has the beneficial effects on the cardiovascular system [9–12].

Flavonoids are considered as the main bioactive compounds of HF, however, the content of flavonoid in HF is rather low (only about 10 mg/g) [13, 14], while the content of sugar is high, which impedes the clinic usage of HF further. If we can get a flavonoid-rich extract from HF, we will make effective utilization of HF. So, the purpose of our research is to get a flavonoid-rich extract from HF, to estimate its pharmacological activities, to find its potential possibility on clinical application. Our previous work showed that the flavonoids found in HF could also be found in the peel of hawthorn fruit (PHF) with higher concentration. In addition, macroporous resin has successfully been applied in the preparative separation of total flavonoids in natural products [15]. In the present study, extract with high content of flavonoids, especially anthocyanin, from the PHF (EPHF) was obtained by Diaion HP-20 adsorption chromatography with 20% aqueous ethanol elution. Phytochemical composition of EPHF was analyzed qualitatively and quantitatively using high performance liquid chromatography (HPLC) and liquid chromatography tandem mass spectrometry (LC-MS). At the same time, the antioxidant capacity, acetylcholinesterase (AChE) inhibitory activity and cytotoxic activity of EPHF were evaluated. To the best of our knowledge, this is the first time to get the extract with rich content of anthocyanin from HF and evaluate its pharmacological activity.

## Methods

### Materials and reagents

Fresh fruit of *Crataegus pinnatifida* Bge. var. *major* N. E. Br. was collected from Beijing China, and identified by Prof. Bin Yang. A voucher specimen (NO. 20160118) was deposited at Institute of Chinese Materia Medica, China Academy of Chinese Medical Sciences. The MCF-7, SKOV-3 cell lines were obtained from the State Key Laboratory of Biotherapy in Sichuan University (Sichuan, China). Cyanidin 3-arabinoside and ideain were purchased from Sigma (St. Louis, MO, USA). Chlorogenic acid, (−)-epicatechin, hyperoside and isoquercitrin were purchased from National Institute for Food and Drug Control (Beijing, China). Procyanidin B2 was purchased from Phytolab (Vestenbergsgreuth, Germany). Diaion HP-20 was purchased from Mitsubishi Chemical Co. Ltd. (Tokyo, Japan). Fluorescein disodium salt (FL), 2,2′-Azobis(2-methylpropionamidine) dihydrochloride (AAPH), 3-(4,5-dimethyl-2-thiazolyl)-2,5-diphenyl-2-H-tetrazolium bromide (MTT), 2,2-Diphenyl- 1-picrylhydrazyl (DPPH), AChE, acetyhhiocholine (ATCI), 6-hydroxy-2,5,7,8- tetramethylchroman-2-carboxylic acid (Trolox), 5,5′-dithiobis- (2-nitrobenzoic acid) (DTNB) were purchased from Sigma (St. Louis, MO, USA). Dulbecco’s modified Eagle’s medium (DMEM), trypsin, penicillin and streptomycin were purchased from HyClone Laboratories Inc., (South Logan, UT, USA). Tris was purchased from Amresco (Solon, OH, USA). HPLC grade acetonitrile and methanol were purchased from Fisher (Fisher Scientific USA). All the other chemicals used from commercial sources were of analytical or higher grade.

### Sample preparation

#### Preparation of EPHF

The peel and flesh of HF were separated and then the peel was lyophilized by lyophilizer (Christ, Germany). The dried PHF was milled into fine powder in a high-speed disintegrator and passed through a 40-mesh sieve before extraction. A sample of 1.0 g of powder was extracted three times with 10 mL of 95% ethanol containing 0.1% (v/v) trifluoroacetic acid at room temperature for each extraction. The whole process of extraction needs to be protected from light. The extract solutions obtained from three extractions were combined, and filtered. Subsequently, the extract solution was concentrated with a vacuum rotary evaporator (Buchi, Swit Zerland) at 40 °C, and then chromatographed on a Diaion HP-20 macroporous resin column, eluted subsequently with water, 20%, and 60% ethanol all containing 0.1% (v/v) trifluoroacetic acid. The fraction eluted by 20% ethanol containing 0.1% (v/v) trifluoroacetic acid was collected, concentrated with a vacuum rotary evaporator and lyophilized.

#### Preparation of the tested sample

The accurately weighed EPHF was dissolved in methanol and filtered by 0.22 μm membrane.

The accurately weighed PHF powder (ca. 1.0 g) was sonicated with 20 mL of 60% ethanol (pH 2) for 10 min at 30 °C, and then centrifuged 10 min at 10,000 rpm, and filtered by 0.22 μm membrane.

#### Preparation of the standard solution

The standards of cyanidin 3-arabinoside, ideain, chlorogenic acid, (−)-epicatechin, hyperoside , isoquercitrin and procyanidin B2 were accurately weighed (ca. 10 mg) , subsequently, dissolved and diluted with methanol.

### Identification of compounds by LC-MS

The analysis was performed on an ultimate 3000 ultra-performance liquid chromatography system coupled to an LTQ Orbitrap mass spectrometer via electrospray ionization (ESI) interface from Thermo Fisher Scientific (Bremen, Germany). This chromatography system consisted of an autosampler, a PDA detector, a column compartment and a quaternary pump. The mass spectra were recorded in simultaneous positive ionization full-scan mode throughout the *m/z* range 50 ~ 800, ion spray voltage 3.5 kV, capillary temperature 350 °C , sheath gas flow rate 50 psi, aux gas flow rate 10 psi.

### Determination of compounds by HPLC

The analysis was performed on an HPLC system (Shimadzu LC-20AT, Tokyo, Japan) consisting of a pump (LC-20A, Tokyo, Japan), a photodiode array detector (SPD-M20A photodiode array detector, Tokyo, Japan), and an Agilent Zorbax Extend C18 column (4.6 × 250 mm, 5 μm) with a solvent flow rate of 0.8 mL/min at 35 °C. Water and acetonitrile both with 2% formic acid were used as the mobile phase A and B, respectively, and the gradient elution program was 5–8% B for 0–5 min, 8–11% B for 5–30 min, 11%–18% B for 30–45 min. Ideain and cyanidin 3-arabinoside were detected at the wavelength of 520 nm, hyperoside and isoquercitrin were detected at the wavelength of 360 nm, (−)-epicatechin was detected at the wavelength of 280 nm, and chlorogenic acid was detected at the wavelength of 330 nm. Data signals were acquired and processed using LC-solution software.

### Pharmacological activity of EPHF

#### Determination of antioxidant capacity

The antioxidant capacities of samples were evaluated by measuring DPPH, AAPH free radical scavenging activity. These two assays were carried out according to our previously reported methods [16, 17]. Trolox was used as positive control in both of the two antioxidant experiments, and the capacity of scavenging AAPH was expressed as Trolox equivalents (μmol Trolox/mg extract).

#### Determination of cell growth inhibition activity

MCF-7 and SKOV-3 cells were cultured in DMEM supplemented with 10% heat inactivated FBS, at 37 °C in a humidified atmosphere of 95% air and 5% CO_2_. The cells were seeded onto 96-well culture plate at a density of 3 × 10^3^ cells/well and allowed to adhere for 24 h. The cells were incubated with different doses (0.5, 5, 12.5, 25 and 50 mg/L) of samples for 48 h. All the reactions were performed in six replicates.

The cell growth inhibition effect of EPHF was evaluated by MTT reduction. After 48 h incubation, MTT solution in PBS was added in a final concentration of 0.5 mg/mL. The plate was incubated at 37 °C for 4 h, followed by the addition of 150 μL DMSO to dissolve the dark blue formazan crystals. The absorbance at 570 nm was measured on a Varioskan Flash Multimode Reader (Thermo Scientific, Finland). Cell viability was calculated as the percentage of untreated control.

#### Determination of AChE inhibitory activity

AChE-inhibiting activities were measured by the slightly modified spectrophotometric method developed by Ellman et al. [18, 19]. Briefly, 10 μL of Tris/HCl buffer containing 20 mM Tris (pH 7.5), 80 μL of DTNB (1 mM), 4 μL of sample, and 4 μL of AChE (2000 U) were mixed in the wells and incubated for 10 min at room temperature. The reaction was initiated by addition of 2 μL of ATCI (20 mM). The microplate was then read at a wavelength of 412 nm every 30 s for 15 min by a Varioskan Flash Multimode Reader (Thermo Scientific, Finland). These samples were dissolved in Tris/HCl buffer (containing 2% MeOH, v/v). All the reactions were performed in six replicates. Galanthamine was used instead of EPHF in positive control group.

## Results and discussion

### The preparation of EPHF

In our previous study, Diaion HP-20 macroporous resin was shown to be a good material to enrich flavonoids in HF [20]. In the present paper, the flavonoids in PHF were enriched using Diaion HP-20 macroporous resin. The sample was eluted with water, 20%, and 60% ethanol all containing 0.1% (v/v) trifluoroacetic acid subsequently, and the corresponding eluents were analyzed by HPLC. The results showed that flavonoids with diversified structures were enriched in 20% ethanol eluent, while sugar was enriched in the fraction eluted by water, and little flavonoids were detected in 60% ethanol eluent. Therefore, the fraction eluted by 20% ethanol was selected for the next experiment.

### Qualitative and quantitative analysis of EPHF

#### Optimization of the sample preparation

To get a high-extraction efficiency of the target compounds from the sample, different extraction solvents (hybrid solutions of ethanol and acid water), the solvent-to-sample ratio, extraction methods (ultrasonic, reflux, and infrared-assisted reflux extraction), temperature (20, 30, 40 and 50 °C) and time (30, 60 and 90 min) were studied. Sixty percent ethanol (pH 2) as the solvent and ultrasonic extraction for 10 min at 30 °C was found to be suitable for the extraction of the target compounds.

#### Qualitative analysis of EPHF

**Fig. 1:**
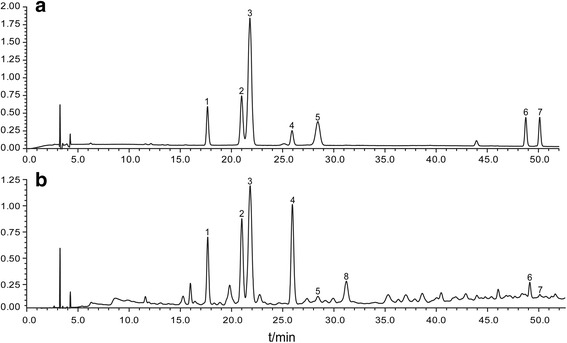
The HPLC chromatograms of reference (**a**) and sample solution (**b**) at 280 nm. Peaks 1 ~ 8: (1) chlorogenic acid; (2) procyanidin B2; (3) ideain; (4) (−)-epicatechin; (5) cyanidin 3-arabinoside; (6) hyperoside; (7) isoquercitrin; (8) procyanidin C1

Chromatographic conditions were optimized for the qualitative analysis of EPHF. In this process, several mobile phases (hybrid systems of organic phase and acid water) were studied, and the best separation result was obtained when using an acetonitrile-formic acid solution as mobile phase at 35 °C (Fig. 1).

Peaks 1 ~ 7 were identified as chlorogenic acid, procyanidin B2, ideain, (−)-epicatechin, cyanidin 3-arabinoside, hyperoside and isoquercitrin by comparing their retention times and UV spectra with those of standard compounds (Fig. 1). Peak 8 was identified as procyanidin C1 according to its precursor ion with an *m/z* value of 867.21 [M + H] ^+^ and other characteristic ions 289.07, 577.13, 579.15, 889.19 [21, 22].

#### Method validation for quantitative analysis

Method validation was performed on parameters such as linearity, recovery and precision. Calibration curves of 6 standard compounds (ideain, (−)-epicatechin, chlorogenic acid, cyanidin 3-arabinoside, hyperoside and isoquercitrin) were constructed from peak areas versus compound amounts. The limit of quantification (LOQ) and limit of detection (LOD) for each compound were determined at signal-to-noise ratios of 10 and 3, respectively. The calibration data were shown in Table 1. Relative standard deviation (RSD) value was calculated, and it was considered as the measure of precision. To assess the intra-day precision, the standard solution was injected seven times within a day. The inter-day precision test was assessed by testing over three consecutive days (Table 2). The assay method precision determination was carried out using six independent sample solutions. The accuracy of quantitation in terms of recovery was assessed. Standard solutions were spiked into the sample solution containing half the mass of the plant material with known amounts of the tested analytes, and the sample was extracted according to the procedure described in sample preparation. The amount of each analyte in the standard solution was almost equal to that in the sample solution. The RSD values of reproducibility were all less than 3%. The average recovery rate of the six analytes ranged from 98.39 to 103.05%, with RSD values varying between 0.73 and 3.60% (Table 2).

**Table 1:** Calibration curves, LODs and LOQs of the standard compounds

Compound	Calibration curve	*r*	Linear range/μg	LOQ/ng	LOD/ng
Ideain	*Y* = 5000000*x*-5992.1	0.9999	0.0049 ~ 0.63	4.93	2.47
Cyanidin 3-arabinoside	*Y* = 5000000*x*-12627	0.9999	0.0049 ~ 0.63	4.89	2.44
Hyperoside	*Y* = 3000000*x*-70021	0.9998	0.0049 ~ 5.02	4.90	2.45
Isoquercitrin	*Y* = 3000000*x*-68963	0.9997	0.0049 ~ 5.01	4.89	2.45
(−)-Epicatechin	*Y* = 835115*x*-7850.7	0.9999	0.0099 ~ 5.12	9.99	2.50
Chlorogenic acid	*Y* = 4000000*x*-131307.7	0.9995	0.0050 ~ 2.57	5.01	2.50

**Table 2:** Precision and recovery for the developed HPLC method

Compound	Precision RSD (%)		Reproducibility	Recovery	
	Intra-day	Inter-day	RSD (%)	Average (%)	RSD (%)
Ideain	1.45	1.45	1.38	98.39	3.60
Cyanidin 3-arabinoside	1.53	1.44	1.74	102.78	1.92
Hyperoside	1.46	1.93	2.12	100.68	0.73
Isoquercitrin	1.44	1.81	0.81	103.05	1.49
(−)-Epicatechin	1.36	1.92	1.72	101.51	3.15
Chlorogenic acid	1.76	1.72	0.34	99.05	0.99

In this paper, the contents of procyanidin B2 and procyanidin C1 in sample were not determined, because of the limited availability of standard of procyanidin C1 and the poor chromatographic separation of procyanidin B2.

#### Quantitative analysis of samples

The contents of the 6 compounds in both of EPHF and PHF were determined, and result was shown in Table 3. The results indicated that the contents of anthocyanin and (−)-epicatechin and the total amount of the 6 compounds in EPHF were much higher than those in PHF. Obviously, the purification method of macroporous resin is useful for the extraction and separation of the flavonoids from PHF.

**Table 3:** Contents of compounds in samples/% (*n = 2*)

Samples	Ideain	Cyanidin 3-arabinoside	Hyperoside	Isoquercitrin	(−)-Epicatechin	Chlorogenic acid	Total
EPHF	17.94	0.14	0.04	0.02	4.09	1.00	23.23
PHF	0.087	0.010	0.038	0.022	0.64	0.084	0.881

*EPHF* extract from the peel of hawthorn fruit, *PHF* peel of hawthorn fruit

### Pharmacological activity of EPHF

#### Antioxidant capacity of EPHF

In this study, the antioxidant capacities of EPHF and PHF were evaluated by measuring DPPH free radical scavenging activity and oxygen radical absorbance capacity (ORAC). The results indicated that EPHF exhibited the strongest DPPH and AAPH free radical scavenging capacity, followed by PHF and HF in a decreasing order [23] (Table 4). Our previous studies have shown that (−)-epicatechin, chlorogenic acid, hyperoside, isoquercitrin and procyanidin B2 all have strong free radical-scavenging activity [16, 17], accordingly, the high concentrations of the antioxidant ingredients in EPHF contributed to the strong antioxidant activity of EPHF, i.e., the higher the concentrations of the antioxidant ingredients, the stronger the antioxidant activity of the sample.

**Table 4:** Free radical scavenging activity of samples (*n = 6*)

	HF	PHF	EPHF	Trolox
DPPH (IC_50_, μg/mL)	330 [17]	66.30	6.72	6.72^a^/8.69^b^
ORAC (μmol/mg)	0.375 ± 0.4 [23]	1.27 ± 0.44	11.65 ± 2.37	

*EPHF* extract from the peel of hawthorn fruit, *PHF* peel of hawthorn fruit, *HF* hawthorn fruit

^a^IC_50_ of Trolox in reference [17]

^b^IC_50_ of Trolox tested in the present experiment

The excessive free radicals in organs may bind to and destroy body cells, and are considered to be the main cause of some diseases, such as aging, cancer and cardiovascular disease [24, 25]. Our results suggest that EPHF may be the potential source of antioxidant agents to be used as an alternative for the treatment of free radical-associated disease.

#### AChE inhibitory activity of EPHF

In this study, the AChE inhibitory activity of EPHF, PHF and HF were evaluated, and the result showed that EPHF exhibited the highest AChE inhibitory activity, followed in decreasing order by PHF and HF (Table 5).

**Table 5:** AChE inhibitory activity of samples (*n = 6*)

	HF	PHF	EPHF	Galanthamine
Anti-AChE (IC_50_, μg/mL)	1385	616.40	11.72	0.29

*EPHF* extract from the peel of hawthorn fruit, *PHF* peel of hawthorn fruit, *HF* hawthorn fruit

AChE plays an important role in the growth and matureness of cell, and it also promotes the development of neurons and the regeneration of nerves. AChE inhibitor has been used as a drug for the symptomatic treatment of Alzheimer’s disease [26]. Our results suggest that EPHF has a relatively strong anti-AChE effect, indicating its potential to be used as an alternative for the treatment of Alzheimer and related disorders.

#### Effect of EPHF on cell growth of human carcinoma

EPHF significantly inhibited MCF-7, SKOV-3 cells proliferation in a concentration-dependent manner with the IC_50_ value of 2.76 and 80.11 μg/mL, respectively. In Li’s study, the IC_50_ value of PHF and hawthorn fruit fleshes on MCF-7 cells were 88.6 and 175.5 μg/mL, respectively [27], which indicated that EPHF might exhibit the stronger inhibitory effect against MCF-7 cells compared with PHF.

In summary, the pharmacological result suggests the potential of EPHF to be an alternative for the treatment of free radical-associated diseases, and Alzheimer and related disorders.

## Conclusions

Macroporous resin is useful for the extraction and separation of the total flavonoids from PHF. The contents of flavonoids especially anthocyanin in EPHF were increased significantly compared with those in PHF and HF. More importantly, the pharmacological experiments showed that EPHF exhibited stronger DPPH and AAPH free radical scavenging activity and AChE inhibitory activity comparing with PHF and HF. Moreover, EPHF inhibited the proliferation of cancer cells. As the first report on the phytochemical compositions and pharmacological activity analysis of EPHF, the results of this study was laying the foundation for further research on the health benefits of hawthorn and its exploitation and utilization.

## Abbreviations

AAPH: 2,2′-Azobis(2-methylpropionamidine) dihydrochloride
AChE: Acetylcholinesterase
ATCI: Acetyhhiocholine
DMEM: Dulbecco’s modified Eagle’s medium
DPPH: 2,2-diphenyl-1-picryhydrazyl
DTNB: 5,5′-dithiobis-(2-nitrobenzoic acid)
EPHF: Extract from the peel of hawthorn fruit
FL: Fluorescein disodium salt
HF: Hawthorn fruit
IC_50_: Half-maximal inhibitory concentration
MTT: 3-(4,5-dimethyl-2-thiazolyl)-2,5-diphenyl-2-H-tetrazolium bromide
ORAC: Oxygen radical absorbance capacity
PHF: Peel of hawthorn fruit
